# Accurate Evaluation and Forecasting in Chemotherapy‐Related Information Needs of People With Breast Cancer: Insights From an Online Medical Consultation Platform

**DOI:** 10.1155/jonm/8640790

**Published:** 2025-12-15

**Authors:** Shuzhi Lin, Qian Liu, Lin Yin, Zimeng Li, Yifang Shen, Bianling Feng

**Affiliations:** ^1^ The Department of Pharmacy Administration and Clinical Pharmacy, School of Pharmacy, Xi’an Jiaotong University, Xi’an, Shaanxi, China, xjtu.edu.cn; ^2^ The Center for Drug Safety and Policy Research, Xi’an Jiaotong University, Xi’an, Shaanxi, China, xjtu.edu.cn

**Keywords:** breast cancer, chemotherapy, healthcare, information needs

## Abstract

**Background:**

Chemotherapy‐related information needs are crucial for patient‐centered care, yet their specific domains remain uncharacterized, particularly lacking large‐scale, high‐quality evidence from real‐world clinical communication contexts.

**Methods:**

The current study used the “Haodf.com” Internet‐based consultation platform. Consultation records of 3000 breast cancer patients were obtained using province‐stratified random sampling with proportional allocation. Using BERTopic modeling and structured data input methods, basic information and chemotherapy‐related information needs of patients were identified. Chi‐square tests and binary logistic regression were used to examine associations between patients’ clinical and demographic characteristics and each information need.

**Results:**

BERTopic modeling initially delineated 10 information need topics; manual structured data entry and validation subsequently uncovered an additional 11 latent needs, resulting in a final taxonomy of 21 need categories. The study enrolled 2781 valid consultation records, from which an average of 2.11 chemotherapy‐related information needs were extracted per record. “Treatment options available,” “benefits and risks of chemotherapy,” “problems of side effects of chemotherapy drugs,” and “current stage of disease and test results” were the top‐ranked information needs. Different information needs were identified and differentiated across specific dimensions, including gender, age, disease metastasis, illness duration, treatment stage, and frequency of interactions.

**Conclusions:**

By creating a quantifiable map of information needs related to breast cancer chemotherapy, the current study established an evidence‐based framework for the creation of precise, patient‐centered, and dynamically adjusted interventional care.

**Highlights**

• Patients with breast cancer had 21 different kinds of information needs connected to chemotherapy.
• Information needs in doctor–patient communication may be precisely identified by the BERTopic model.
• Variables like gender, age, metastasis, illness duration, treatment stage, and interaction frequency affect information needs.
• The study offers a starting point for creating patient‐focused and dynamically adaptable demand intervention strategies.

## 1. Introduction

Breast cancer is the second most prevalent disease diagnosis worldwide, with 2.3 million new cases reported in 2022 [1]. According to projections, there will be 3.2 million new cases of breast cancer by 2050 [2]. The 5‐year survival statistics for breast cancer have improved recently because of advancements in early screening, medical technology, and new medicine development. The reported 5‐year survival rate for localized stage cancer varies between 87% and 89.6% [3, 4] in countries or regions with comparatively high medical standards, such as the United States, Singapore, Turkey, and Hong Kong. In China, the age‐standardized 5‐year survival rate for breast cancer reached 80.9% between 2019 and 2021 [5].

As survival rates improve and diagnoses occur at younger ages [2, 6], more survivors now prioritize quality of life over longevity. A large number of studies have actively advocated a patient‐centered approach to cancer diagnosis and treatment [7–9], which focuses more on the needs of patients and treatment preferences. Thus, a progressive replacement of the conventional doctor‐led, disease‐centered service approach is occurring. However, a previous study reported that patients have positive attitudes toward receiving disease‐related knowledge, although their needs are not currently met [10]. Several studies have shown that the unmet supportive care needs of cancer patients are concentrated in the domain health system and information [11–14]. Higher information needs may increase patient anxiety [15] and can have a negative impact on overall quality of life [14, 16].

A previous study predicted that breast cancer will account for 12.7% of chemotherapy cases by 2040, ranking second, and that China will have the greatest demand for chemotherapy of any country [17]. Although chemotherapy significantly reduces the chance of breast cancer recurrence and increases overall patient survival [18, 19], it also involves psychological and physical difficulties, which can lead to an increase in patient needs [20]. Patients who receive care that addresses their information needs may be better able to handle challenging circumstances [21]. Focus groups, questionnaire assessments, and systematic reviews have all been used in previous studies to thoroughly examine the information needs of breast cancer survivors [11, 16, 22, 23]. However, most existing studies have elaborated the information needs from the macro perspective of disease and overall treatment, and few studies have been conducted on the level of chemotherapy needs, especially in the Chinese population.

Survey techniques like questionnaire evaluation are vulnerable to sample bias, access restrictions, and patient recollection bias. The Internet has emerged as one of the main options for patients seeking information in a time when their understanding of empowerment and self‐management is steadily growing [24]. Thus, accurately extracting and mining the information needs of patients with breast cancer regarding chemotherapy from consultation platform data are important for developing efficient strategies to offer individualized nursing services.

Topic modeling, an unsupervised machine learning method, constitutes a core technique in natural language processing (NLP) and text mining [25]. The method can identify possible topic themes from vast volumes of unstructured data and does not require preexisting labels for manual classification or training datasets. In the field of topic modeling, the BERTopic model, which is based on the Transformer architecture, demonstrates unique advantages, which can automatically iterate and determine the optimal number of topics. Notably, the model demonstrates significant performance improvements in capturing complex semantic and contextual information in text, enabling more effective understanding and processing of deep semantic relationships within text [25].

Therefore, the main goal of the current study was to identify the information needs related to chemotherapy for breast cancer patients and their variability among different groups. The secondary goal was to explore the potential of topic modeling to identify information needs in consultation records.

## 2. Methods

### 2.1. Data Sources

Haodf.com is one of the earliest and most widely known Internet‐based medical platforms in China [26] and was selected as the data source in this study. As of September 2024, 280,000 doctors have registered with their real names on Haodf.com, and the cumulative number of patients served exceeds 89 million [27].

### 2.2. Data Extraction

**Figure 1:**
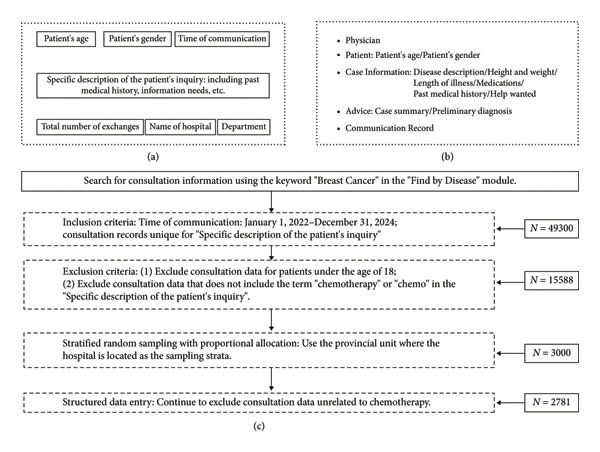
Schematic diagram of data extraction. Note: (a) the brief consultation record (crawling allowed); (b) the detail page of each consultation record (crawling not allowed); and (c) flow diagram of inclusion/exclusion criteria for consultation records.

The study gathered comprehensive consultation records using the keyword “Breast Cancer” in the “Find by Disease” module of the Haodf.com Internet‐based medical platform. Each consultation record is sorted according to the time of communication on the search page, which displays only brief consultation records (as illustrated in Figure 1(a)). The collection of consultation information was conducted using web crawler technology in the Python programming language. The crawler program accurately extracted the link to the detail page of each consultation record and seven critical pieces of information shown in Figure 1(a), which were used to determine further inclusion and exclusion criteria.

The study covered consultation data from January 1, 2022, to December 31, 2024. To maximally ensure record independence, only the earliest consultation time entry was retained whenever the crawler program detected that the “Specific description of the patient’s inquiry” field was duplicated.

Records for patients aged < 18 years old were excluded. As the researchers examined the detail pages of the extracted consultation records (Figure 1(b)) for breast cancer in detail, they found that some consultation records, such as those that exclusively involved surgery and endocrine therapy, did not include information about chemotherapy. Therefore, the study further omitted consultation records that did not include the word “chemotherapy” or “chemo” in the “Specific description of the patient’s inquiry” to maximize the extraction of chemotherapy‐related consultation records (Figure 1(c)).

Stratified random sampling with proportional allocation was carried out on available consultation records that met the inclusion and exclusion criteria, with the provincial administrative unit where the consulting hospitals are located serving as the sampling strata. The provincial sample was sized in proportion to the available consultation records, and cases were then selected at random within each province. To balance data representativeness and the practicality of manual data entry, a total of 3000 consultation records were sampled. Supporting 1 shows the sampling results for each province.

### 2.3. Study Design

Step 1: Topic modeling.

Figure 1(b) shows the detail page of each consultation record, where patients usually state their needs directly in the “Help Wanted” section. As web crawling of detail pages was prohibited, manual extraction of the complete “Help Wanted” text for all 3000 consultation records was deemed infeasible under our personnel and time constraints. We, therefore, selected 1400 consultation records by simple random sampling without replacement and manually extracted the complete “Help Wanted” text for topic modeling. We adopted the BERTopic model for topic modeling, and its results will provide guidance for the subsequent entry of information needs. Detailed parameters of the model are presented in Table 1.

**Table 1:** Parameter settings for BERTopic topic modeling.

Steps	Parameter name	Parameter settings
Step1: Embeddings: BERT	embedding_model	Paraphrase‐multilingual‐MiniLM‐L12‐v2

Step2: Dimensionality reduction: UMAP	n_neighbors	15
	n_component	5
	min_dist	0.00
	Metric	Cosine
	random_state	15

Step3: Clustering: HDBSCAN	min_cluster_size	10
	min_samples	5
	Metric	Euclidean

This study used the Jieba Chinese word segmentation engine in the Python language environment as the basic tool. A custom vocabulary strategy was used to address the fragmentation issues that may arise during word segmentation in specific contexts. At the same time, considering the diversity of Chinese language expression, a synonym dictionary was constructed to improve the comprehensiveness and accuracy of semantic understanding. Stopword processing was based on “hit_stopwords” [28] and expanded according to the research context.

On the basis of the clustering results of hierarchical clustering structure, intertopic distance map, and topic similarity matrix, we combined disciplinary background to delete and merge topics, gradually reducing the number of topics, and ultimately obtaining a definitive set of topic categories. We used c‐TF‐IDF to extract and optimize the keywords of the requirements and selected the top 10 feature words with the highest c‐TF‐IDF values as representative keywords.

Step 2: Manual structured data entry and validation.

The study recruited seven master’s degree entrants to form an eight‐person entry team with the project leader (Shuzhi Lin). The entry team was responsible for data entry and initial organization, and all team members possessed a solid foundation of professional knowledge, with professional backgrounds covering nursing, pharmacy administration, clinical medicine, and other fields.

The project leader presampled 10% of the consultation records for use in developing entry criteria for consultation records. In this study, the following four sections of information were extracted from the content in the detail page (Figure 1(b)) of each consultation record: (1) patient demographics: gender, age, height, and weight of the patient; (2) disease progression and treatment: metastasis, duration of illness, and stage of treatment; (3) characteristics of the consultation: total number of exchanges during the current consultation; and (4) core content of the consultation: patients’ information needs related to chemotherapy. It should be noted that the contents of (1) and (3) were already extracted in the crawler session of “2.2 Data extraction” and only needed to be verified by the entrant. The information in (2) and (4) should be considered together with the “case information” and “communication record” in the detail page (Figure 1(b)).

Prior to the formal entry, the project leader carried out group training several times to ensure that the entry team personnel were proficient in the entry process and entry standards. During the formal entry stage, when entry team members encountered new information needs that did not fall under the results of the BERTopic modeling in Step 1, they immediately contacted the project leader. When a new need was defined, the project leader convened a meeting to communicate the information to all entry staff.

We continued to exclude consultation records unrelated to chemotherapy in the step of the data entry phase. Each consultation record was independently entered into a predefined Excel template by two entry personnel according to the same criteria, and entries that were not agreed upon were verified and revised by the project leader.

### 2.4. Statistical Analysis

Categorical variables were described using frequencies and percentages. All information needs identified after structured data entry (Step 2 in Section 2.2) were sorted in descending order based on their occurrence frequency. To screen for core needs, we initially selected the top‐ranked information needs that accounted for more than 80% of the cumulative proportion of total need instances. Meanwhile, significant jumps in the proportion of patients with information needs were also taken into consideration. The core information needs will be included in the subsequent analysis of influencing factors.

We used listwise deletion to handle missing data. To explore the associations between each information need and patients’ characteristics (including demographic information, disease progress and treatment, and consultation characteristics), univariable analyses were conducted using the Chi‐square test (with Yates’ continuity correction where appropriate). Variables with *p* ≥ 0.10 in the univariable analyses across all information needs were excluded from subsequent multivariable analyses.

Multivariable analyses were performed using the binary logistic regression model. When complete separation occurred in the dependent variable, Firth’s penalized logistic regression was employed instead. For the multivariable analyses, each information need was treated as a dependent variable (1 = presence of the need and 0 = absence of the need). Odds ratios (ORs) and their corresponding 95% confidence intervals (95% CIs) were used to quantify the strength of associations between patients’ characteristics and each information need.

*p* < 0.05 was considered statistically significant. Excel 2019 (Microsoft Corporation, Redmond, WA, USA), R Version 4.5.1 (R Foundation for Statistical Computing, Vienna, Austria) and IBM SPSS Statistics Version 26.0 (IBMCorp., Armonk, N.Y., USA) were used for statistical analysis.

### 2.5. Ethical Approval

Approval for this study was received from the medicine and biomedical ethics committee of Xi’an Jiaotong University (No. 2024–1942).

## 3. Results

### 3.1. Results of Topic Modeling

**Figure 2:**
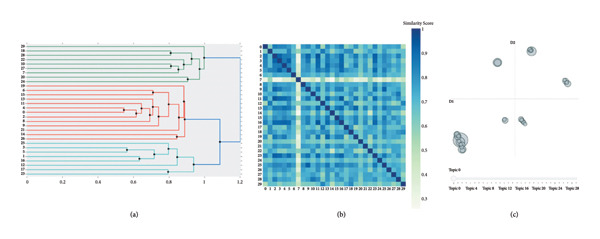
Clustering results for information need topics (*n* = 1400). (a) Hierarchical clustering, (b) similarity matrix, and (c) intertopic distance map.

The study used the BERTopic model to preliminarily identify 30 information need topics (Topic 0–Topic 29, details in Supporting 2). On the basis of the clustering results shown in Figure 2 and the disciplinary background, the original topics were deleted (Topic 17, Topic 18, and Topic 29) and merged. The final classification of chemotherapy information needs for breast cancer patients was divided into the following 10 topics (T0–T9), sorted by the number of text counts they covered from highest to lowest (Table 2): treatment options available, benefits and risks of chemotherapy, chemotherapy drug dosage, frequency and cycles, problems of metastasis and relapse, current stage of disease and test results, level of treatment by doctor/hospital, traditional Chinese medicine treatment, problems of side effects of chemotherapy drugs, dietary guidance, and appointment scheduling for chemotherapy.

**Table 2:** Distribution of topics and topic keywords related to chemotherapy information needs of breast cancer patients based on the BERTopic model (*n* = 1400).

Serial number	Text count	Research topic	Topic keywords	Merged content
T0	425	Treatment options available	Breast cancer, drug regimen, chemotherapy, treatment, surgery, counseling, follow‐up, postoperative, radiotherapy, metastasis	Topic 0, Topic 1, Topic 3, Topic 5, Topic 7
T1	281	Benefits and risks of chemotherapy	Chemotherapy, necessity, whether, treatment, drug regimen, programmatic, need, situation, follow‐up, genetic testing	Topic 2, Topic 4, Topic 8, Topic 9, Topic 11, Topic 14, Topic 16, Topic 21
T2	60	Chemotherapy drug dosage, frequency and cycles	Number of times, chemotherapy, programmatic, drug regimen, treatment, endocrine, radiotherapy, follow‐up, currently, counseling	Topic 13, Topic 15, Topic 25
T3	57	Problems of metastasis and relapse	Bone metastases, lung, surgery, chest wall, metastasis, treatment, lesion, examination, axilla, need	Topic 10, Topic 24, Topic 28
T4	42	Current stage of disease and test results	Surgery, come out, whether, positive, resection, normal, biopsy, test, none, necessity	Topic 22, Topic 26, Topic 27
T5	42	Level of treatment by doctor/hospital	Beijing, surgery, Shanghai, hospital, whether, examination, chemotherapy, when, need, treatment	Topic 6
T6	27	Herbal medicine conditioning traditional Chinese medicine treatment	Medication, counseling, drug regimen, regulation, oral administration, traditional Chinese medicine, this time, obtained, today, medicines	Topic 12
T7	17	Problems of side effects of chemotherapy drugs	Liver function, hepatoprotective drugs, liver, hepatitis C, liver disease, next time, treatment, reinforcement, today, metastasis	Topic 20
T8	17	Dietary guidance	Chemotherapy, recipes, several models, number of times, excessive, third, fifth, synchronized, persistence, treatment course	Topic 19
T9	15	Appointment scheduling for chemotherapy	Chemotherapy, work, arrange, malignant, unfavorable, as soon as possible, epidemic, continue, situation, counseling	Topic 23

### 3.2. Patients’ Basic Information

The entry team excluded 219 consultation records unrelated to chemotherapy after careful reading, and the total number of consultation records finally included in the statistical analysis was 2781. Among them (Table 3), the proportion of female patients was higher than that of male patients, accounting for 98.89% of consultation records. Only 31 consultation records (1.11%) were recorded for male patients. Regarding age, more than 30% of the patients were in the 50–59 years age group (31.90%), followed by the 40–49 years age group (25.78%). Body mass index (BMI) information was unavailable for 116 individuals. Among the 2665 patients with reliable BMI data, more than half (57.97%) of the patients had a BMI value in the normal range, 29.31% were overweight, and 7.88% were obese.

**Table 3:** Basic information about breast cancer patients in the consultation records (*n* = 2781).

Patient information	Item	Category	Number	Percentage (%)
Patient demographics	Gender	Female	2750	98.89
		Male	31	1.11
	Age (years)	< 30	66	2.37
		30–39	532	19.13
		40–49	717	25.78
		50–59	887	31.90
		60–69	471	16.94
		≥ 70	108	3.88
	BMI^a^	Underweight < 18.5	129	4.84
		Normal 18.5–23.9	1545	57.97
		Overweight 24–27.9	781	29.31
		Obese ≥ 28	210	7.88

Disease progression and treatment	Disease transfer	Yes	790	28.41
		No	610	21.93
		Not yet clear	1381	49.66
	Duration of illness^b^	Within 1 month	834	30.81
		1–3 months	262	9.68
		3–6 months	808	29.85
		More than 6 months	803	29.66
	Stage of treatment	Have not started treatment	151	5.43
		Treatment other than chemotherapy	938	33.73
		Already receiving chemotherapy	1692	60.84

Characteristics of the consultation	Total number of exchanges	< 10	894	32.15
		10–29	1381	49.66
		≥ 30	506	18.19

^a^Data missing for 116 patients; percentages calculated from 2665 valid cases.

^b^Data missing for 74 patients; percentages calculated from 2707 valid cases.

A total of 1400 records (50.34%) of the included consultation records were for patients whose disease metastasis status could be definitively diagnosed, of which 790 patients had metastasis and 610 had no metastasis. The metastatic disease status of patients in the remaining 1381 (49.66%) consultation records was unclear. For 74 individuals, information on the duration of illness was not accessible. Among the remaining 2707 patients, the duration of illness was mainly concentrated in “within 1 month (30.81%),” “3–6 months (29.85%),” and “more than 6 months (29.66%).” Regarding the stage of treatment, 151 patients (5.43%) had not yet started treatment, 938 patients (33.73%) were in the stage of having started treatment but not yet undergoing chemotherapy, and 1692 patients (60.84%) had received chemotherapy. The overall number of exchanges in the consultation was primarily focused in the 10–29 range (49.66%). Furthermore, the percentage of consultation records with less than 10 total exchanges was 32.15%, but the percentage of consultation records with 30 or more total exchanges was only 18.19%.

### 3.3. Types of Information Needs

In the present study, an analysis of 2781 consultation records revealed 21 categories of information needs with a total of 5857 instances (Table 4), averaging 2.11 per patient. Ranked first in terms of frequency was the information need related to “Treatment Options Available,” which was present in 1887 consultation records (32.22% of the total needs), indicating its prominent prevalence among patients’ inquiries. In addition, “Benefits and Risks of Chemotherapy” and “Problems of Side Effects of Chemotherapy Drugs” also ranked highly, accounting for 12.26% and 10.77% of the total demand, further highlighting the high level of concern about the negative effects of access to chemotherapy. While focusing on the details of medication, patients also showed a high desire to know more about the type of disease, disease progression and prognosis.

**Table 4:** Information need categories and proportions.

Types of information needs	Number	Percentage (%, *n* = 5857)	Cumulative percentage (%, *n* = 5857)	Patient‐based proportion (%, *n* = 2781)
**Treatment options available**	1887	32.22	32.22	67.85
**Benefits and risks of chemotherapy**	718	12.26	44.48	25.82
**Problems of side effects of chemotherapy drugs**	631	10.77	55.25	22.69
**Current stage of disease and test results**	569	9.71	64.96	20.46
**Problems of metastasis and relapse**	473	8.08	73.04	17.01
**Chemotherapy drug dosage, frequency, and cycles**	350	5.98	79.02	12.59
Survival and prognosis	272	4.64	83.66	9.78
Effectiveness/efficacy of existing chemotherapy regimens	260	4.44	88.10	9.35
**Treatment standards of doctors/hospitals**	145	2.48	90.58	5.21
**Dietary guidance**	109	1.86	92.44	3.92
**Traditional Chinese medicine treatment**	91	1.55	93.99	3.27
Differences between domestically produced and imported drugs	89	1.52	95.51	3.20
Treatment costs	84	1.43	96.94	3.02
Precautions during recovery	69	1.18	98.12	2.48
**Appointment scheduling for chemotherapy**	51	0.87	98.99	1.83
Drug clinical trial issues	28	0.48	99.47	1.01
Fertility issues	15	0.26	99.73	0.54
Chemotherapy infusion equipment issues	7	0.12	99.85	0.25
Chemotherapy drug purchasing issues	7	0.12	99.97	0.25
Genetic issues	1	0.02	99.98	0.04
Drug resistance of treatment regimens	1	0.02	100.00	0.04

*Note:* Categories in bold correspond to the information needs identified by topic modeling in Section 3.1 (Table 2).

Compared with the results of BERTopic modeling in Section 3.1, “Survival and Prognosis” was a new and highly ranked information need that emerged during the data entry process, and the demand for “Problems of Side Effects of Chemotherapy Drugs” significantly rose in the ranking. The 10 information need categories identified by BERTopic modeling covered 85.78% of the total 5857 information need instances (derived from data entry).

### 3.4. Investigation of Factors Associated With Information Needs

After excluding medical consultation records in which duration of illness or BMI was recorded as “unknown” (both variables include an “unknown” entry option, as shown in Table 3), a total of 2596 valid records (see Supporting 3 for demographic characteristics) were used for the further analysis. The 7 information needs accounted for a cumulative 83.66% of total instances, and noting the notable drop in patient‐based proportion between Need 8 (9.35%) and Need 9 (5.21%), we confirmed these 8 needs as the final core information needs for subsequent analyses. The results of the 8 chi‐square tests (Supporting 4) showed that there was no significant association between BMI values and any of the core information *n* needs. Therefore, in the subsequent logistic regression, only the remaining six variables were included (gender, age, disease transfer, duration of illness, stage of treatment, and total number of exchanges).

Logistic regression results (Table 5) showed that male patients were more than three times as likely as female patients to report needs about the benefits and risks of chemotherapy (OR: 3.73 and 95% CI: 1.46–9.54) and survival and prognosis (OR: 3.35 and 95% CI: 1.36–8.26), whereas female patients were significantly more likely to express information needs regarding available treatment options.

**Table 5:** Multivariable binary logistic regression analysis of patients’ clinical and demographic characteristics and each information need (*n* = 2596).

Item	Category	Need 1		Need 2		Need 3		Need 4		Need 5		Need 6		Need 7		Need 8^a^	
		OR (95% CI)	*P*	OR (95% CI)	*P*	OR (95% CI)	*P*	OR (95% CI)	*P*	OR (95% CI)	*P*	OR (95% CI)	*P*	OR (95% CI)	*P*	OR (95% CI)	*P*
Gender	Female	1	**0.041**	1	**0.006**	1	0.531	1	0.053	1	0.155	1	0.874	1	**0.009**	1	0.250
	Male	0.43 (0.20–0.97)		3.73 (1.46–9.54)		1.33 (0.54–3.28)		2.21 (0.99–4.93)		1.92 (0.78–4.68)		0.91 (0.27–3.09)		3.35 (1.36–8.26)		1.99 (0.58–5.62)	
Age (years)	< 30	1	0.088	1	**0.005**	1	**0.029**	1	0.847	1	**0.003**	1	0.839	1	0.471	1	0.346
	30–39	0.79 (0.42–1.47)		0.82 (0.40–1.65)		1.02 (0.53–1.99)		1.03 (0.51–2.07)		0.65 (0.35–1.21)		0.84 (0.39–1.81)		0.80 (0.34–1.88)		2.85 (0.91–14.23)	
	40–49	0.74 (0.40–1.37)		1.07 (0.54–2.13)		0.94 (0.49–1.81)		1.20 (0.61–2.38)		0.46 (0.25–0.85)		0.81 (0.38–1.73)		0.57 (0.24–1.34)		2.58 (0.83–12.86)	
	50–59	0.65 (0.35–1.20)		1.09 (0.55–2.17)		1.39 (0.73–2.65)		1.22 (0.62–2.4)		0.43 (0.23–0.80)		0.97 (0.46–2.05)		0.81 (0.35–1.87)		2.16 (0.70–10.75)	
	60–69	0.56 (0.30–1.06)		1.23 (0.61–2.48)		1.36 (0.70–2.66)		1.15 (0.57–2.31)		0.68 (0.36–1.29)		0.83 (0.38–1.82)		0.72 (0.30–1.72)		2.81 (0.88–14.14)	
	≥ 70	0.51 (0.25–1.07)		2.48 (1.09–5.62)		1.24 (0.55–2.78)		1.33 (0.59–2.98)		0.46 (0.21–1.03)		0.71 (0.26–1.89)		0.68 (0.24–1.93)		3.22 (0.83–17.79)	
Disease transfer	Yes	1	**< 0.001**	1	**0.001**	1	**< 0.001**	1	0.514	1	**< 0.001**	1	0.113	1	**< 0.001**	1	**0.001**
	No	0.57 (0.43–0.75)		1.82 (1.34–2.48)		1.62 (1.21–2.16)		1.08 (0.81–1.44)		1.30 (0.97–1.74)		1.09 (0.78–1.53)		0.66 (0.45–0.95)		0.85 (0.57–1.26)	
	Not yet clear	0.35 (0.28–0.44)		1.53 (1.16–2.01)		1.70 (1.34–2.15)		1.15 (0.91–1.45)		0.75 (0.58–0.96)		0.81 (0.61–1.09)		0.50 (0.36–0.69)		0.57 (0.42–0.78)	
Duration of illness	Within 1 month	1	0.264	1	**0.003**	1	**< 0.001**	1	0.097	1	0.087	1	**< 0.001**	1	0.926	1	**< 0.001**
	1–3 months	0.75 (0.54–1.04)		1.00 (0.68–1.46)		1.05 (0.74–1.50)		1.07 (0.73–1.56)		1.11 (0.74–1.65)		0.88 (0.58–1.33)		0.85 (0.50–1.44)		1.64 (0.99–2.72)	
	3–6 months	1.01 (0.79–1.29)		0.87 (0.66–1.14)		0.86 (0.66–1.12)		1.25 (0.95–1.64)		0.95 (0.70–1.29)		0.83 (0.61–1.12)		0.92 (0.63–1.32)		1.20 (0.80–1.82)	
	More than 6 months	1.00 (0.77–1.29)		0.57 (0.42–0.78)		0.47 (0.35–0.63)		1.41 (1.06–1.86)		1.34 (0.99–1.81)		0.31 (0.21–0.46)		0.92 (0.63–1.35)		0.57 (0.36–0.89)	
Stage of treatment	Have not started treatment	1	**< 0.001**	1	**< 0.001**	1	**< 0.001**	1	**0.008**	1	0.539	1	0.318	1	**< 0.001**	1	**< 0.001**
	Treatment other than chemotherapy	0.71 (0.44–1.17)		2.01 (1.37–2.97)		2.21 (1.13–4.34)		0.65 (0.43–0.99)		0.76 (0.47–1.24)		1.63 (0.87–3.07)		0.77 (0.46–1.28)		5.67 (0.76–723.75)	
	Already receiving chemotherapy	0.28 (0.17–0.45)		0.18 (0.12–0.27)		5.61 (2.88–10.92)		0.53 (0.35–0.81)		0.78 (0.48–1.26)		1.55 (0.82–2.91)		0.35 (0.21–0.59)		46.98 (6.77–5932.61)	
Total number of exchanges	< 10	1	0.577	1	0.459	1	**< 0.001**	1	**< 0.001**	1	**< 0.001**	1	**< 0.001**	1	**< 0.001**	1	**0.019**
	10–29	1.10 (0.91–1.34)		0.87 (0.68–1.10)		1.45 (1.15–1.81)		1.70 (1.34–2.15)		2.64 (1.98–3.51)		1.43 (1.06–1.92)		1.98 (1.38–2.82)		1.11 (0.80–1.54)	
	≥ 30	1.02 (0.79–1.31)		0.96 (0.70–1.31)		1.96 (1.48–2.58)		2.41 (1.81–3.20)		3.77 (2.72–5.24)		2.33 (1.66–3.29)		2.77 (1.83–4.17)		1.67 (1.14–2.44)	

*Note:* Need 1: treatment options available; Need 2: benefits and risks of chemotherapy; Need 3: problems of side effects of chemotherapy drugs; Need 4: current stage of disease and test results; Need 5: problems of metastasis and relapse; Need 6: chemotherapy drug dosage, frequency, and cycles; Need 7: survival and prognosis; Need 8: effectiveness/efficacy of existing chemotherapy regimens. The bold values represent *p* < 0.05.

^a^Due to the problem of complete separation in the “Stage of treatment” group for Need 8, Firth’s penalized logistic regression was used.

Age was a significant predictor of three information needs: benefits and risks of chemotherapy, problems of side effects of chemotherapy drugs, and problems of metastasis and relapse. In particular, patients aged ≥ 70 years were 2.48 times more likely to ask about the benefits and risks of chemotherapy than patients younger than 30 years old. Compared with patients aged 40–59, those under 30 years old were more focused on problems of metastasis and relapse.

Patients with no metastasis and unknown metastatic status were more concerned about the benefits and risks of chemotherapy and the problems of side effects of chemotherapeutic drugs, whereas patients with clear metastasis were more likely to be concerned about treatment options available, survival and prognosis. Patients with unknown metastatic status were less concerned about the problems of metastasis and relapse and the effectiveness/efficacy of existing chemotherapy regimens than patients with known metastatic status. Duration of illness exerted a significant effect on four information needs (Need 2, Need 3, Need 6, and Need 8). Compared with patients with a disease duration of 1 month, patients with a disease duration of more than 6 months were less concerned about the benefits and risks of chemotherapy (OR: 0.57 and 95% CI: 0.42–0.78), the problems of the side effects of chemotherapy drugs (OR: 0.47 and 95% CI: 0.35–0.63), chemotherapy drug dosage, frequency, and cycles (OR: 0.31 and 95% CI: 0.21–0.46), and effectiveness/efficacy of existing chemotherapy regimens (OR: 0.57 and 95% CI: 0.36–0.89).

Patients who had not yet started treatment were more interested in information about the treatment options available, the current stage of disease and test results, survival, and prognosis. Patients who started treatment but did not undergo chemotherapy were the most concerned about the benefits and risks of chemotherapy. At the same time, the likelihood of patients asking questions about the problems of side effects of chemotherapy drugs and the effectiveness/efficacy of existing chemotherapy regimens at the time of consultation increased significantly as treatment progressed. The number of exchanges had a significant effect on six of the eight information needs, with the exception of the treatment options available and the benefits and risks of chemotherapy.

## 4. Discussion

The current national sampling survey revealed that breast cancer patients often have unmet chemotherapy‐related information needs. The analysis further broke down these needs into 21 clear categories and revealed significant differences among different patient groups in eight information needs, providing a basis for targeted information support strategies to improve patients’ quality of life. Additionally, the analysis results confirmed that BERTopic modeling can reliably identify and categorize the information needs of breast cancer patients, providing methodological feasibility evidence for its application in tumor support treatment research.

When patients feel that the information provided by healthcare providers during communication is insufficient or questionable, they often use Internet‐based platforms to seek a “second opinion” [29]. Online interactive healthcare can address patients’ information and emotional needs [30]. Therefore, in this study, the frequent occurrence of information needs such as chemotherapy regimens, treatment benefits and drawbacks, side effects, and disease test results indicates that these areas are key points of unmet needs among breast cancer patients, which is consistent with the results of previous studies [10, 16, 31–34]. Additionally, the results revealed that “Traditional Chinese medicine treatment” is a unique phenomenon in the information needs of Chinese breast cancer patients. This need was not mentioned in previous studies conducted in other countries.

In the current study, the “survival and prognosis” demand was identified during the information entry phase rather than the BERTopic modeling phase. Given the differences in the content covered by the two stages, it was determined that this need was more prominently reflected in the “communication record.” At the same time, the results of binary logistic regression also confirmed that as the consultation gradually deepened, patients were more likely to express a need for information about survival rates and prognosis. In this study, only two types of information needs, “treatment options available” and “benefits and risks of chemotherapy” were not affected by the number of consultations and were the focus of widespread patient concern.

A previous study reported that cancer patients face high levels of economic toxicity during treatment [35]. Understanding specific information about treatment costs can help patients better plan their financial expenditure. However, in our study, the demand for information about “treatment costs” ranked low (accounting for only 1.43%). This may because China has centralized government negotiations and procurement of cancer drugs and included more cancer drugs into the catalog of medical insurance reimbursement [36], which effectively reduces the medication burden on breast cancer patients [37]. Additionally, the barriers to entry and cost structures of online consultation platforms exhibit certain selective characteristics. Private platforms like Haodf.com, with their higher fees [26] and requirements for users’ digital literacy, tend to attract users with stronger economic foundations and higher levels of digital literacy.

In the current study, the chemotherapy‐related needs of breast cancer patients change dynamically with factors such as disease stage and treatment progress, and there are significant differences in the focus of needs at different stages. Previous studies [16, 33, 34, 38] have also found that unmet supportive care needs of breast cancer patients are significantly associated with demographic factors and disease factors. For example, research by Davinia et al. revealed that patients recently diagnosed with metastatic breast cancer exhibited a high demand for information [16]. Halbach et al. found that patients had a high and rising demand for information (e.g., side effects, drug information, medical examination results, and treatment plans) in the first 10 weeks after breast cancer surgery [33]. Hongru Lu et al. reported that patients’ demand for information at different stages of their breast cancer journey was regulated by a variety of variables [34].

This study involved several limitations. First, since the data have been fully deidentified and identifiers such as patient IDs have been removed, we cannot determine whether there are multiple consultation records from the same patient. This may cause bias in the analysis results of affecting factors. Meanwhile, the study is unable to capture the dynamic trajectory of individuals’ information needs across the chemotherapy cycle. Second, the study only enabled the categorization of the types of demands that patients had during consultations, not measurement of the scope of those needs. Finally, although family support was present in a relatively small proportion of consultations, the engagement of family members may have resulted in a distorted expression of the needs of the patients, potentially introducing bias into the study outcomes.

## 5. Conclusions

The BERTopic model and structured data entry methods were used in this study to comprehensively analyze the categories of chemotherapy‐related information needs of Chinese breast cancer patients using Internet consultation records. Factors such as gender, illness metastasis, and treatment stage all exerted a dynamic effect on information demands. The findings of this study not only provide new perspectives for a comprehensive understanding of chemotherapy‐related information needs in breast cancer patients but they also provide important evidence‐based guidance for healthcare professionals to develop personalized information services and accurately implement future intervention trials.

## Ethics Statement

Approval for this study was received from the medicine and biomedical ethics committee of Xi’an Jiaotong University (No. 2024‐1942).

## Disclosure

All authors read and approved the final manuscript.

## Conflicts of Interest

The authors declare no conflicts of interest.

## Author Contributions

Conceptualization and methodology: Bianling Feng and Shuzhi Lin. Formal analysis: Shuzhi Lin. Data curation: Shuzhi Lin, Qian Liu, and Lin Yin. Visualization: Zimeng Li and Yifang Shen. Supervision and writing–review and editing: Bianling Feng. Writing–original draft: Shuzhi Lin.

## Funding

No funding was received for this manuscript

## Supporting Information

Additional supporting information can be found online in the Supporting Information section.

Table S1. STROBE statement: Checklist of items that should be included in reports of cross‐sectional studies.

## Supporting information


**Supporting Information 1** Supporting 1: In this study, a total of 3000 consultation records were sampled. Supporting 1 shows, for each province, the sample sizes obtained by stratified random sampling with proportional allocation and the corresponding numbers of consultation records finally entered into the analysis.

**Supporting Information 2** Supporting 2: Supporting 2 presents the distribution of the 30 topics and their characteristic words initially identified by the BERTopic model used in this study.

**Supporting Information 3** Supporting 3: This supporting summarizes the demographic information of the breast cancer patients constituting the final valid sample for the “Analysis of factors associated with information needs” in Section 3.4.

**Supporting Information 4** Supporting 4: summarizes chi‐square test results linking information needs (e.g., Treatment Options Available, Benefits and Risks of Chemotherapy, Problems of Side Effects of Chemotherapy Drugs, Current Stage of Disease and Test Results, Problems of Metastasis, Relapse, Chemotherapy Drug Dosage, Frequency and Cycles, Survival and Prognosis, Effectiveness/efficacy of existing chemotherapy regimens) to variables such as gender, age, BMI, and disease‐related factors. These relationships highlight how patient demographics and conditions contribute to differences in information‐seeking priorities.

## Data Availability

The data that support the findings of this study are available from the corresponding author upon reasonable request.
