# Supplementary material for: Accurate Evaluation and Forecasting in Chemotherapy‐Related Information Needs of People With Breast Cancer: Insights From an Online Medical Consultation Platform
Source: J Nurs Manag. 2025 Dec 15;2025:8640790. doi: 10.1155/jonm/8640790 (PMC12714160; doi:10.1155/jonm/8640790)
Supplement: Supplementary file 1 — Supporting Information 1 Supporting 1: In this study, a total of 3000 consultation records were sampled. Supporting 1 shows, for each province, the sample sizes obtained by stratified random sampling with proportional allocation and the corresponding numbers of consultation records finally entered into the analysis. [file JONM-2025-8640790-s002.docx]

Supplementary 1: In this study, a total of 3,000 consultation records were sampled. Supplementary 1 shows, for each province, the sample sizes obtained by stratified random sampling with proportional allocation and the corresponding numbers of consultation records finally entered into the analysis.


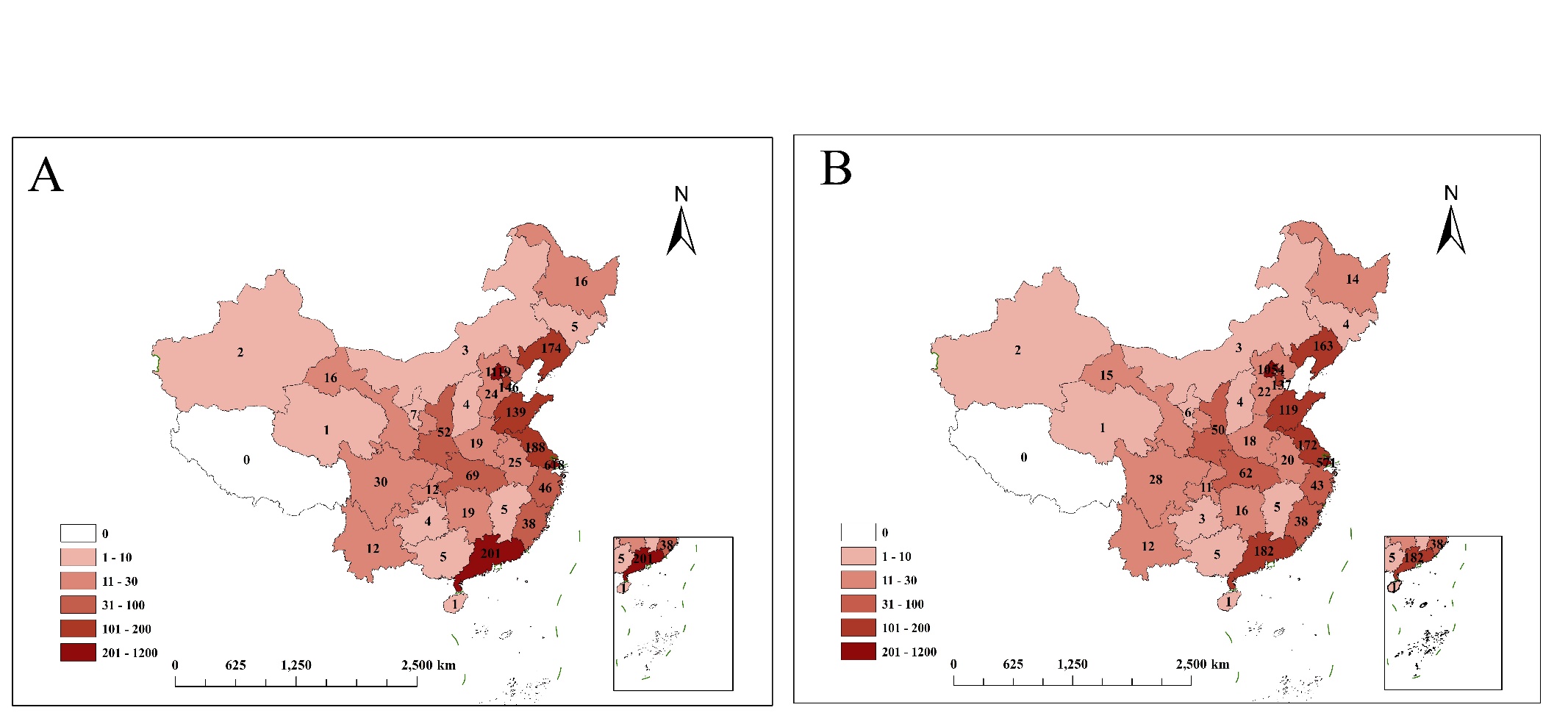


Figure 1 Schematic diagram of stratified random sampling with proportional allocation and final entry results of consultation records by province

Note: A: Distribution chart of stratified random sampling with proportional allocation in various provinces of China; B: Final inclusion numbers for consultation records in various provinces of China. China map content approval number GS (2024) 0650.
