# Supplementary material for: Accurate Evaluation and Forecasting in Chemotherapy‐Related Information Needs of People With Breast Cancer: Insights From an Online Medical Consultation Platform
Source: J Nurs Manag. 2025 Dec 15;2025:8640790. doi: 10.1155/jonm/8640790 (PMC12714160; doi:10.1155/jonm/8640790)
Supplement: Supplementary file 2 — Supporting Information 2 Supporting 2: Supporting 2 presents the distribution of the 30 topics and their characteristic words initially identified by the BERTopic model used in this study. [file JONM-2025-8640790-s003.docx]

Supplementary 2: Supplementary 2 presents the distribution of the 30 topics and their characteristic words initially identified by the BERTopic model used in this study.

Table 1 Topic-Characteristic Word Distribution for 30 Information Needs Topics

| Topic | Text Count | Characteristic Words |
| --- | --- | --- |
| Topic0 | 201 | breast cancer, surgery, chemotherapy, metastases, postoperative, breast, breast-conserving, treatment, left breast, right breast |
| Topic1 | 77 | control, disease status, next step, drug regimen, follow-up, optimal, treatment, seek, fever, counseling |
| Topic2 | 72 | necessity, chemotherapy, postoperative, treatment, vascular, chemoradiotherapy, nerve, surgery, conservative, possible |
| Topic3 | 67 | situation, comparison, chemotherapy, benefit, total mastectomy, suitability, treatment, body, use, trastuzumab deruxtecan |
| Topic4 | 57 | programmatic, number of times, whether, chemotherapy, suggestion, drug regimen, need, now, radiotherapy, genetic testing |
| Topic5 | 44 | hope, drug regimen, counseling, suggestion, chemoradiotherapy, endocrine, include, treatment, follow-up, specific |
| Topic6 | 42 | Beijing, Shanghai, hospital, surgery, time, test, whether, as soon as possible, chemotherapy, need |
| Topic7 | 36 | now, comprehensive, clear, question, programmatic, add number, situation, pay attention, feasible, examination |
| Topic8 | 35 | situation, comparison, chemotherapy, benefit, total mastectomy, suitability, treatment, body, use, trastuzumab deruxtecan |
| Topic9 | 31 | need, finish, chemotherapy, now, whether, don't want, capecitabine, surgery, invasive, follow-up |
| Topic10 | 31 | bone metastases, chest wall, MRI, suspect, rib, examination, scan, suggest, pain, consider |
| Topic11 | 30 | programmatic, necessity, chemotherapy, obvious, treatment, whether, change, targeted therapy, immediately, twice |
| Topic12 | 27 | medication, regulation, counseling, traditional Chinese medicine, oral administration, this time, drug regimen, today, ask |
| Topic13 | 26 | number of times, programmatic, paclitaxel, opinion, endocrine, doxorubicin, drug regimen, cyclophosphamide, chemotherapy, subtype |
| Topic14 | 24 | genetic testing, exemption, immunohistochemistry, risk, score, drug regimen, mucinous, pure, necessity, recommendation |
| Topic15 | 20 | time, number of times, finish, chemotherapy, one week, continue, advance, stop medication, need, uterus |
| Topic16 | 17 | one step, if needed, roughly, should, hepatitis C, chemotherapy, necessity, drug regimen, therapy, need |
| Topic17 | 17 | hope, help, better, neratinib, as soon as possible, give, provide, in-hospital, buy medicine, Jingzhou |
| Topic18 | 17 | goserelin, anastrozole, oral, no need, abdominal injection, endocrine, radiotherapy, lymph nodes, injection, need |
| Topic19 | 17 | recipes, several varieties, excessive, third, five cycles, synchronized, persist, chemotherapy, treatment course, tamoxifen |
| Topic20 | 17 | liver function, hepatoprotective drugs, liver, hepatitis C, liver disease, next time, enhance, today, feeding, delay |
| Topic21 | 15 | elderly, age, must, body, patient, measures, chemoradiotherapy, flow, hope, immunity |
| Topic22 | 15 | surgery, triple-negative, breast-conserving, not feasible, on-site, quickly, invasive, right now, which date, understand |
| Topic23 | 15 | work, arrange, malignant, poor, as soon as possible, epidemic, give, chemotherapy, continue, fatigue |
| Topic24 | 14 | lung, lesion, cough, cardiopulmonary, lobular, willing, lung metastasis, infection, confirmed diagnosis, cause |
| Topic25 | 14 | Second time, number of times, hope, one year, add single, hyperthyroidism, significant, intermittent, hold on, difference |
| Topic26 | 14 | come out, positive, negative, test, listen, biopsy, targeted therapy, hospital, consult, immunotherapy |
| Topic27 | 13 | normal, comparison, images, sudden, cancer onset, conclusion, newly emerged, last year, summary, attached separately |
| Topic28 | 12 | axilla, armpit, clearance, lymph, bulge, lymph nodes, symptomatic treatment, surgery, contralateral, biopsy |
| Topic29 | 10 | taking, abemaciclib, switch medication, reduce, sleep, two years, Western medicine, dosage, letrozole, poor |
