# Supplementary material for: Accurate Evaluation and Forecasting in Chemotherapy‐Related Information Needs of People With Breast Cancer: Insights From an Online Medical Consultation Platform
Source: J Nurs Manag. 2025 Dec 15;2025:8640790. doi: 10.1155/jonm/8640790 (PMC12714160; doi:10.1155/jonm/8640790)
Supplement: Supplementary file 3 — Supporting Information 3 Supporting 3: This supporting summarizes the demographic information of the breast cancer patients constituting the final valid sample for the “Analysis of factors associated with information needs” in Section 3.4. [file JONM-2025-8640790-s004.docx]

Supplementary 3: This supplementary summarizes the demographic information of the breast cancer patients constituting the final valid sample for the “Analysis of factors associated with information needs” in Section 3.4.

Table 1 Demographic Characteristics of the Breast Cancer Patients Included in the Information Needs Factor Analysis

| Patient Information | Item | Category | Number | Percentage (%, n=2596) |
| --- | --- | --- | --- | --- |
| Patient demographics | Gender | Female | 2569 | 98.96 |
|  |  | Male | 27 | 1.04 |
|  | Age (years) | ＜30 | 62 | 2.39 |
|  |  | 30-39 | 495 | 19.07 |
|  |  | 40-49 | 677 | 26.08 |
|  |  | 50-59 | 817 | 31.47 |
|  |  | 60-69 | 444 | 17.10 |
|  |  | ≥70 | 101 | 3.89 |
|  | BMI | Underweight <18.5 | 126 | 4.85 |
|  |  | Normal 18.5-23.9 | 1504 | 57.94 |
|  |  | Overweight 24-27.9 | 761 | 29.31 |
|  |  | Obese ≥28 | 205 | 7.90 |
| Disease progression and treatment | Disease Transfer | Yes | 741 | 28.54 |
|  |  | No | 575 | 22.15 |
|  |  | Not yet clear | 1280 | 49.31 |
|  | Duration of illness | Within 1 month | 785 | 30.24 |
|  |  | 1-3 months | 237 | 9.13 |
|  |  | 3-6 months | 795 | 30.62 |
|  |  | More than 6 months | 779 | 30.01 |
|  | Stage of treatment | Have not started treatment | 134 | 5.16 |
|  |  | Treatment other than chemotherapy | 879 | 33.86 |
|  |  | Already receiving chemotherapy | 1583 | 60.98 |
| Characteristics of the consultation | Total number of exchanges | ＜10 | 823 | 31.70 |
|  |  | 10-29 | 1298 | 50.00 |
|  |  | ≥30 | 475 | 18.30 |

Note: The 2,596 breast cancer patients in this table constitute the final valid sample for the “Analysis of factors associated with information needs” presented in Section 3.4.
