# Supplementary material for: Accurate Evaluation and Forecasting in Chemotherapy‐Related Information Needs of People With Breast Cancer: Insights From an Online Medical Consultation Platform
Source: J Nurs Manag. 2025 Dec 15;2025:8640790. doi: 10.1155/jonm/8640790 (PMC12714160; doi:10.1155/jonm/8640790)
Supplement: Supplementary file 4 — Supporting Information 4 Supporting 4: summarizes chi‐square test results linking information needs (e.g., Treatment Options Available, Benefits and Risks of Chemotherapy, Problems of Side Effects of Chemotherapy Drugs, Current Stage of Disease and Test Results, Problems of Metastasis, Relapse, Chemotherapy Drug Dosage, Frequency and Cycles, Survival and Prognosis, Effectiveness/efficacy of existing chemotherapy regimens) to variables such as gender, age, BMI, and disease‐related factors. These relationships highlight how patient demographics and conditions contribute to differences in information‐seeking priorities. [file JONM-2025-8640790-s001.docx]

Supplementary 4: summarizes chi-square test results linking information needs (e.g., Treatment Options Available, Benefits and Risks of Chemotherapy, Problems of Side Effects of Chemotherapy Drugs, Current Stage of Disease and Test Results, Problems of Metastasis, Relapse, Chemotherapy Drug Dosage, Frequency and Cycles, Survival and Prognosis, Effectiveness/efficacy of existing chemotherapy regimens) to variables such as gender, age, BMI, and disease-related factors. These relationships highlight how patient demographics and conditions contribute to differences in information-seeking priorities.

Table 1 Chi-square test statistics for information needs and related variables

| **Item** | ***P* value** | | | | | | | |
| --- | --- | --- | --- | --- | --- | --- | --- | --- |
|  | **Need1** | **Need2** | **Need3** | **Need4** | **Need5** | **Need6** | **Need7** | **Need8** |
| **Gender** | 0.075 | 0.002 | 0.675 | 0.037 | 0.324^a^ | 1.000^a^ | 0.009^a^ | 0.518^a^ |
| **Age** | 0.542 | <0.001 | 0.086 | 0.834 | 0.006 | 0.671 | 0.417 | 0.428 |
| **BMI** | 0.342 | 0.231 | 0.224 | 0.785 | 0.185 | 0.865 | 0.119 | 0.480 |
| **Disease Transfer** | <0.001 | <0.001 | 0.001 | 0.993 | <0.001 | 0.012 | <0.001 | <0.001 |
| **Duration of illness** | <0.001 | <0.001 | <0.001 | 0.492 | 0.084 | <0.001 | 0.028 | <0.001 |
| **Stage of treatment** | <0.001 | <0.001 | <0.001 | 0.062 | 0.868 | 0.007 | <0.001 | <0.001 |
| **Total number of exchanges** | 0.111 | 0.223 | <0.001 | <0.001 | <0.001 | <0.001 | <0.001 | <0.001 |

Note: a means Yates’s correction for continuity. Need1: Treatment Options Available; Need2: Benefits and Risks of Chemotherapy; Need3: Problems of Side Effects of Chemotherapy Drugs; Need4: Current Stage of Disease and Test Results; Need5: Problems of Metastasis and Relapse; Need6: Chemotherapy Drug Dosage, Frequency and Cycles; Need7: Survival and Prognosis; Need8: Effectiveness/efficacy of existing chemotherapy regimens.

Table 2 Cross-Tabulations of Explanatory Variables and Information Needs [n (%)]

| **Item** | **Category** | **Need1** | | **Need2** | | **Need3** | | **Need4** | | **Need5** | | **Need6** | | **Need7** | | **Need8** | | **Total** |
| --- | --- | --- | --- | --- | --- | --- | --- | --- | --- | --- | --- | --- | --- | --- | --- | --- | --- | --- |
|  |  | absence | presence | absence | presence | absence | presence | absence | presence | absence | presence | absence | presence | absence | absence | absence | presence |  |
| **Gender** | **Female** | 823(32.00%) | 1746(68.00%) | 1912(74.40%) | 657(25.60%) | 1990(77.50%) | 579(22.50%) | 2039(79.40%) | 530(20.60%) | 2135(83.10%) | 434(16.90%) | 2248(87.50%) | 321(12.50%) | 2330(90.70%) | 239(9.30%) | 2330(90.70%) | 239(9.30%) | 2569(100.00%) |
|  | **Male** | 13(48.10%) | 14(51.90%) | 13(48.10%) | 14(51.90%) | 20(74.10%) | 7(25.90%) | 17(63.00%) | 10(37.00%) | 20(74.10%) | 7(25.90%) | 24(88.90%) | 3(11.10%) | 20(74.10%) | 7(25.90%) | 23(85.20%) | 4(14.80%) | 27(100.00%) |
|  | **Total** | 836(32.20%) | 1760(67.80%) | 1925(74.20%) | 671(25.80%) | 2010(77.40%) | 586(22.60%) | 2056(79.20%) | 540(20.80%) | 2155(83.00%) | 441(17.00%) | 2272(87.50%) | 324(12.50%) | 2350(90.50%) | 246(9.50%) | 2353(90.60%) | 243(9.40%) | 2596(100.00%) |
| **Age** | **＜30** | 16(25.80%) | 46(74.20%) | 45(72.60%) | 17(27.40%) | 49(79.00%) | 13(21.00%) | 51(82.30%) | 11(17.70%) | 46(74.20%) | 16(25.80%) | 53(85.50%) | 9(14.50%) | 55(88.70%) | 7(11.30%) | 60(96.80%) | 2(3.20%) | 62(100.00%) |
|  | **30-39** | 151(30.50%) | 344(69.50%) | 386(78.00%) | 109(22.00%) | 386(78.00%) | 109(22.00%) | 400(80.80%) | 95(19.20%) | 395(79.80%) | 100(20.20%) | 431(87.10%) | 64(12.90%) | 445(89.90%) | 50(10.10%) | 441(89.10%) | 54(10.90%) | 495(100.00%) |
|  | **40-49** | 209(30.90%) | 468(69.10%) | 504(74.40%) | 173(25.60%) | 546(80.60%) | 131(19.40%) | 533(78.70%) | 144(21.30%) | 575(84.90%) | 102(15.10%) | 597(88.20%) | 80(11.80%) | 627(92.60%) | 50(7.40%) | 611(90.30%) | 66(9.70%) | 677(100.00%) |
|  | **50-59** | 273(33.40%) | 544(66.60%) | 613(75.00%) | 204(25.00%) | 606(74.20%) | 211(25.80%) | 641(78.50%) | 176(21.50%) | 700(85.70%) | 117(14.30%) | 705(86.30%) | 112(13.70%) | 732(89.60%) | 85(10.40%) | 747(91.40%) | 70(8.60%) | 817(100.00%) |
|  | **60-69** | 153(34.50%) | 291(65.50%) | 322(72.50%) | 122(27.50%) | 342(77.00%) | 102(23.00%) | 354(79.70%) | 90(20.30%) | 354(79.70%) | 90(20.30%) | 395(89.00%) | 49(11.00%) | 401(90.30%) | 43(9.70%) | 402(90.50%) | 42(9.50%) | 444(100.00%) |
|  | **≥70** | 34(33.70%) | 67(66.30%) | 55(54.50%) | 46(45.50%) | 81(80.20%) | 20(19.80%) | 77(76.20%) | 24(23.80%) | 85(84.20%) | 16(15.80%) | 91(90.10%) | 10(9.90%) | 90(89.10%) | 11(10.90%) | 92(91.10%) | 9(8.90%) | 101(100.00%) |
|  | **Total** | 836(32.20%) | 1760(67.80%) | 1925(74.20%) | 671(25.80%) | 2010(77.40%) | 586(22.60%) | 2056(79.20%) | 540(20.80%) | 2155(83.00%) | 441(17.00%) | 2272(87.50%) | 324(12.50%) | 2350(90.50%) | 246(9.50%) | 2353(90.60%) | 243(9.40%) | 2596(100.00%) |
| **BMI** | **18.5-23.9** | 494(32.80%) | 1010(67.20%) | 1121(74.50%) | 383(25.50%) | 1147(76.30%) | 357(23.70%) | 1190(79.10%) | 314(20.90%) | 1269(84.40%) | 235(15.60%) | 1313(87.30%) | 191(12.70%) | 1370(91.10%) | 134(8.90%) | 1354(90.00%) | 150(10.00%) | 1504(100.00%) |
|  | **＜18.5** | 47(37.30%) | 79(62.70%) | 102(81.00%) | 24(19.00%) | 94(74.60%) | 32(25.40%) | 96(76.20%) | 30(23.80%) | 102(81.00%) | 24(19.00%) | 112(88.90%) | 14(11.10%) | 115(91.30%) | 11(8.70%) | 116(92.10%) | 10(7.90%) | 126(100.00%) |
|  | **24-27.9** | 229(30.10%) | 532(69.90%) | 552(72.50%) | 209(27.50%) | 606(79.60%) | 155(20.40%) | 609(80.00%) | 152(20.00%) | 616(80.90%) | 145(19.10%) | 670(88.00%) | 91(12.00%) | 689(90.50%) | 72(9.50%) | 699(91.90%) | 62(8.10%) | 761(100.00%) |
|  | **＞28** | 66(32.20%) | 139(67.80%) | 150(73.20%) | 55(26.80%) | 163(79.50%) | 42(20.50%) | 161(78.50%) | 44(21.50%) | 168(82.00%) | 37(18.00%) | 177(86.30%) | 28(13.70%) | 176(85.90%) | 29(14.10%) | 184(89.80%) | 21(10.20%) | 205(100.00%) |
|  | **Total** | 836(32.20%) | 1760(67.80%) | 1925(74.20%) | 671(25.80%) | 2010(77.40%) | 586(22.60%) | 2056(79.20%) | 540(20.80%) | 2155(83.00%) | 441(17.00%) | 2272(87.50%) | 324(12.50%) | 2350(90.50%) | 246(9.50%) | 2353(90.60%) | 243(9.40%) | 2596(100.00%) |
| **Disease Transfer** | **Yes** | 164(22.10%) | 577(77.90%) | 627(84.60%) | 114(15.40%) | 608(82.10%) | 133(17.90%) | 588(79.40%) | 153(20.60%) | 597(80.60%) | 144(19.40%) | 651(87.90%) | 90(12.10%) | 647(87.30%) | 94(12.70%) | 641(86.50%) | 100(13.50%) | 741(100.00%) |
|  | **No** | 150(26.10%) | 425(73.90%) | 348(60.50%) | 227(39.50%) | 446(77.60%) | 129(22.40%) | 455(79.10%) | 120(20.90%) | 449(78.10%) | 126(21.90%) | 483(84.00%) | 92(16.00%) | 512(89.00%) | 63(11.00%) | 529(92.00%) | 46(8.00%) | 575(100.00%) |
|  | **Not yet clear** | 522(40.80%) | 758(59.20%) | 950(74.20%) | 330(25.80%) | 956(74.70%) | 324(25.30%) | 1013(79.10%) | 267(20.90%) | 1109(86.60%) | 171(13.40%) | 1138(88.90%) | 142(11.10%) | 1191(93.00%) | 89(7.00%) | 1183(92.40%) | 97(7.60%) | 1280(100.00%) |
|  | **Total** | 836(32.20%) | 1760(67.80%) | 1925(74.20%) | 671(25.80%) | 2010(77.40%) | 586(22.60%) | 2056(79.20%) | 540(20.80%) | 2155(83.00%) | 441(17.00%) | 2272(87.50%) | 324(12.50%) | 2350(90.50%) | 246(9.50%) | 2353(90.60%) | 243(9.40%) | 2596(100.00%) |
| **Duration of illness** | **Within 1 month** | 204(26.00%) | 581(74.00%) | 437(55.70%) | 348(44.30%) | 614(78.20%) | 171(21.80%) | 632(80.50%) | 153(19.50%) | 658(83.80%) | 127(16.20%) | 654(83.30%) | 131(16.70%) | 690(87.90%) | 95(12.10%) | 745(94.90%) | 40(5.10%) | 785(100.00%) |
|  | **1-3 months** | 94(39.70%) | 143(60.30%) | 175(73.80%) | 62(26.20%) | 166(70.00%) | 71(30.00%) | 191(80.60%) | 46(19.40%) | 195(82.30%) | 42(17.70%) | 201(84.80%) | 36(15.20%) | 217(91.60%) | 20(8.40%) | 201(84.80%) | 36(15.20%) | 237(100.00%) |
|  | **3-6 months** | 273(34.30%) | 522(65.70%) | 634(79.70%) | 161(20.30%) | 584(73.50%) | 211(26.50%) | 629(79.10%) | 166(20.90%) | 676(85.00%) | 119(15.00%) | 684(86.00%) | 111(14.00%) | 730(91.80%) | 65(8.20%) | 690(86.80%) | 105(13.20%) | 795(100.00%) |
|  | **More than 6 months** | 265(34.00%) | 514(66.00%) | 679(87.20%) | 100(12.80%) | 646(82.90%) | 133(17.10%) | 604(77.50%) | 175(22.50%) | 626(80.40%) | 153(19.60%) | 733(94.10%) | 46(5.90%) | 713(91.50%) | 66(8.50%) | 717(92.00%) | 62(8.00%) | 779(100.00%) |
|  | **Total** | 836(32.20%) | 1760(67.80%) | 1925(74.20%) | 671(25.80%) | 2010(77.40%) | 586(22.60%) | 2056(79.20%) | 540(20.80%) | 2155(83.00%) | 441(17.00%) | 2272(87.50%) | 324(12.50%) | 2350(90.50%) | 246(9.50%) | 2353(90.60%) | 243(9.40%) | 2596(100.00%) |
| **Stage of treatment** | **Have not started treatment** | 22(16.40%) | 112(83.60%) | 86(64.20%) | 48(35.80%) | 124(92.50%) | 10(7.50%) | 96(71.60%) | 38(28.40%) | 109(81.30%) | 25(18.70%) | 122(91.00%) | 12(9.00%) | 112(83.60%) | 22(16.40%) | 134(100.00%) | 0(0.00%) | 134(100.00%) |
|  | **Treatment other than chemotherapy** | 192(21.80%) | 687(78.20%) | 387(44.00%) | 492(56.00%) | 733(83.40%) | 146(16.60%) | 692(78.70%) | 187(21.30%) | 731(83.20%) | 148(16.80%) | 745(84.80%) | 134(15.20%) | 763(86.80%) | 116(13.20%) | 861(98.00%) | 18(2.00%) | 879(100.00%) |
|  | **Already receiving chemotherapy** | 622(39.30%) | 961(60.70%) | 1452(91.70%) | 131(8.30%) | 1153(72.80%) | 430(27.20%) | 1268(80.10%) | 315(19.90%) | 1315(83.10%) | 268(16.90%) | 1405(88.80%) | 178(11.20%) | 1475(93.20%) | 108(6.80%) | 1358(85.80%) | 225(14.20%) | 1583(100.00%) |
|  | **Total** | 836(32.20%) | 1760(67.80%) | 1925(74.20%) | 671(25.80%) | 2010(77.40%) | 586(22.60%) | 2056(79.20%) | 540(20.80%) | 2155(83.00%) | 441(17.00%) | 2272(87.50%) | 324(12.50%) | 2350(90.50%) | 246(9.50%) | 2353(90.60%) | 243(9.40%) | 2596(100.00%) |
| **Total number of exchanges** | **＜10** | 287(34.90%) | 536(65.10%) | 607(73.80%) | 216(26.20%) | 674(81.90%) | 149(18.10%) | 703(85.40%) | 120(14.60%) | 755(91.70%) | 68(8.30%) | 751(91.30%) | 72(8.70%) | 779(94.70%) | 44(5.30%) | 758(92.10%) | 65(7.90%) | 823(100.00%) |
|  | **10-29** | 396(30.50%) | 902(69.50%) | 951(73.30%) | 347(26.70%) | 1000(77.00%) | 298(23.00%) | 1011(77.90%) | 287(22.10%) | 1047(80.70%) | 251(19.30%) | 1135(87.40%) | 163(12.60%) | 1161(89.40%) | 137(10.60%) | 1188(91.50%) | 110(8.50%) | 1298(100.00%) |
|  | **≥30** | 153(32.20%) | 322(67.80%) | 367(77.30%) | 108(22.70%) | 336(70.70%) | 139(29.30%) | 342(72.00%) | 133(28.00%) | 353(74.30%) | 122(25.70%) | 386(81.30%) | 89(18.70%) | 410(86.30%) | 65(13.70%) | 407(85.70%) | 68(14.30%) | 475(100.00%) |
|  | **Total** | 836(32.20%) | 1760(67.80%) | 1925(74.20%) | 671(25.80%) | 2010(77.40%) | 586(22.60%) | 2056(79.20%) | 540(20.80%) | 2155(83.00%) | 441(17.00%) | 2272(87.50%) | 324(12.50%) | 2350(90.50%) | 246(9.50%) | 2353(90.60%) | 243(9.40%) | 2596(100.00%) |

Note: Need1: Treatment Options Available; Need2: Benefits and Risks of Chemotherapy; Need3: Problems of Side Effects of Chemotherapy Drugs; Need4: Current Stage of Disease and Test Results; Need5: Problems of Metastasis and Relapse; Need6: Chemotherapy Drug Dosage, Frequency and Cycles; Need7: Survival and Prognosis; Need8: Effectiveness/efficacy of existing chemotherapy regimens.
